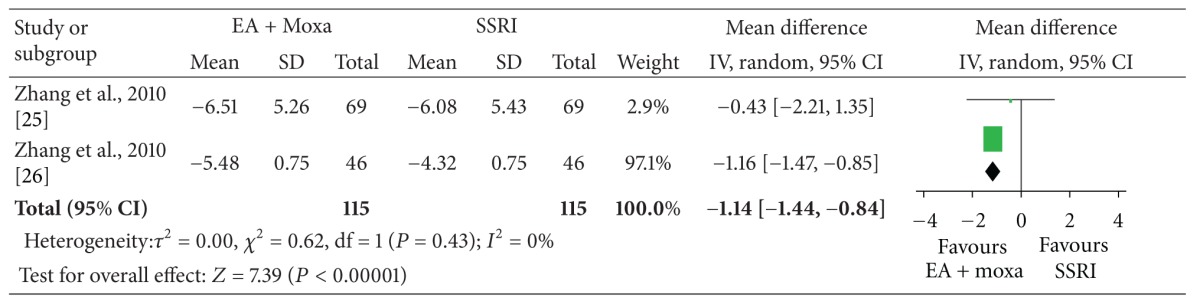# Acupuncture for Posttraumatic Stress Disorder: A Systematic Review of Randomized Controlled Trials and Prospective Clinical Trials

**DOI:** 10.1155/2013/615857

**Published:** 2013-02-06

**Authors:** Young-Dae Kim, In Heo, Byung-Cheul Shin, Cindy Crawford, Hyung-Won Kang, Jung-Hwa Lim

**Affiliations:** ^1^School of Korean Medicine, Pusan National University, Yangsan 626-870, Republic of Korea; ^2^Division of Clinical Medicine, School of Korean Medicine, Pusan National University, Yangsan 626-870, Republic of Korea; ^3^Samueli Institute, 1737 King Street, Suite 600, Alexandria, VA 22314, USA; ^4^Department of Neuropsychiatry & Herbal Resources, Professional Graduate School of Korean Medicine, Won-Kwang University, Iksan 570-749, Republic of Korea; ^5^Department of Neuropsychiatry, Korean Medical Hospital, Pusan National University, Yangsan 626-789, Republic of Korea

## Abstract

To evaluate the current evidence for effectiveness of acupuncture for posttraumatic stress disorder (PTSD) in the form of a systematic review, a systematic literature search was conducted in 23 electronic databases. Grey literature was also searched. The key search terms were “*acupuncture*” and “*PTSD.*” No language restrictions were imposed. We included all randomized or prospective clinical trials that evaluated acupuncture and its variants against a waitlist, sham acupuncture, conventional therapy control for PTSD, or without control. Four randomized controlled trials (RCTs) and 2 uncontrolled clinical trials (UCTs) out of 136 articles in total were systematically reviewed. One high-quality RCT reported that acupuncture was superior to waitlist control and therapeutic effects of acupuncture and cognitive-behavioral therapy (CBT) were similar based on the effect sizes. One RCT showed no statistical difference between acupuncture and selective serotonin reuptake inhibitors (SSRIs). One RCT reported a favorable effect of acupoint stimulation plus CBT against CBT alone. A meta-analysis of acupuncture plus moxibustion versus SSRI favored acupuncture plus moxibustion in three outcomes. This systematic review and meta-analysis suggest that the evidence of effectiveness of acupuncture for PTSD is encouraging but not cogent. Further qualified trials are needed to confirm whether acupuncture is effective for PTSD.

## 1. Introduction

Posttraumatic stress disorder (PTSD) develops following a stressful event or situation of an exceptionally threatening or catastrophic nature, which is likely to cause pervasive distress [1]. PTSD is classified as an anxiety disorder and is typically defined by the coexistence of 3 clusters of symptoms, namely, *reexperiencing*, *marked avoidance*, and *hyperarousal *[2]. The prevalence rates of PTSD have been reported as 6–25% [3], and approximately 25–30% of people experiencing a traumatic event may go on to develop PTSD [4]. 

Current first-line PTSD therapies include trauma-focused cognitive behavioral therapy (CBT), stress inoculation training, and pharmacotherapies [5]. Complementary and alternative medicine (CAM) interventions include a range of therapies that are not considered standard to the practice of medicine in the USA. CAM therapies are widely used by mental health consumers, including veterans, and numerous stakeholders have expressed strong interest in fostering the evidence base for these approaches in PTSD [6]. In addition, approximately 21% of CAM users met diagnostic criteria for at least one problematic mental disorder, according to one study [7]. 

Acupuncture is commonly recognized worldwide as a mainstream CAM therapy. Acupuncture is the practice of inserting a needle or needles into certain points in the body, known as meridian acupuncture points, for therapeutic or preventive purposes [8]. Numerous studies have shown that acupuncture is well tolerated by patients, safe, and cost effective compared to routine care [9]. 

Additionally, acupuncture is widely used in mental disorders such as anxiety disorders [10], dementia [11], eating disorders [12], schizophrenia [13], sleep disorders [14], and substance-related disorders [15, 16]. Electroacupuncture is effective in rat models of stress and thus might be a useful adjunct therapy in stress-related anxiety disorders [17]. Acupuncture has positive effects in PTSD patients, although the evidence is still lacking as to its true efficacy for this condition [18]. 

There have been two reviews published on acupuncture or its variants for PTSD [19, 20]. David Feinstein reviewed 2 randomized controlled trials (RCTs) and 6 outcome studies which tested whether brief psychological exposure with acupoint tapping was effective for PTSD or not and its conclusion was not confirmative [19]. Also Michael Hollifield reviewed acupuncture for PTSD referring one published and one unpublished clinical trial and suggested further definitive research is needed because of lack of well-conducted RCTs [20]. However, there has been no systematic review published to date fully summarizing the current total evidence about the quality and effectiveness of acupuncture for PTSD. For this reason, we conducted a systematic review of RCTs and prospective clinical trials to assess critically whether acupuncture improves the symptoms of PTSD and to make recommendations for future research based on gap areas identified in this review.

## 2. Methods

### 2.1. Data Sources and Search Strategy

Following the COSI model [21], we searched the following electronic databases over time periods from their inception to July 2012: Cochrane Database of Systematic Reviews, the Cochrane Central Register of Controlled Trials (CENTRAL), MEDLINE through PubMed, EMBASE, Allied and Complementary Medicine Database (AMED), CINAHL, Pilots, Google, Korean databases (which include DBpia, Korea Institute of Science and Technology Information (KISTI), KoreaMed, Korean traditional knowledge portal, OASIS, RISS, the National Assembly Library, and The National Library of Korea), Chinese databases (which include China Academic Journal, http://www.cqvip.com and WANFANG DATA), and a Japanese database (Japan Science and Technology Information Aggregator Electronic). We also searched the grey literature; unpublished trials were searched via the Register of the Controlled Trials databases ( http://www.controlled-trials.com and http://www.clinicaltrials.gov), and we communicated with identified experts in the field of acupuncture and PTSD, searched our departmental files, and pearled the references of all included articles for other relevant articles perhaps not picked up through other methods of searching.

The key search terms were “(acupuncture OR acup*) AND (stress disorders, post-Traumatic OR posttraumatic stress disorder OR posttraumatic stress disorder OR PTSD).” MeSH strategy was applied to ensure the most powerful search where applicable. Search strategies were adjusted for each of the databases. Personal contacts were made with the original authors of the searched studies to identify any potential missing data from the publications. 

### 2.2. Study Selection

Two psychiatrists (J. H. Lim and H. W. Kang) actively participated in the study selection process based on clinical expertise, and two experienced researchers (B. C. Shin, C. Cindy) monitored the whole process of systematic review. All reviewers were fully trained in the systematic review process executed. 

#### 2.2.1. Types of Studies

The review was not restricted by study design, however, study should be prospective clinical trials. We included RCTs and nonrandomized controlled trials that compared acupuncture or its variants with a control or control groups. We also included uncontrolled clinical trials (UCTs) of acupuncture for PTSD to give our research question a more solid ground or to make recommendations for future research. However, we separately analyzed RCTs and others, and interpreted more weighted on RCTs because of research quality following the validity of evidence. No restrictions were imposed on studies with regard to blinding, languages, or year published.

#### 2.2.2. Types of Participants

We selected all studies including patients with PTSD diagnosed by any set of criteria, DSM-IV or ICD-10, regardless of gender, age, nationality, or outpatient therapy or inpatient therapy. 

#### 2.2.3. Types of Interventions/Controls

Clinical trials investigating any type of needling acupuncture, specifically classical acupuncture, electroacupuncture, auricular acupuncture were included. We also included trials that included acupuncture as a more complex intervention, that is, acupuncture plus another intervention if the comparison group was that other intervention. We included trials using control groups with no treatment, sham/placebo acupuncture, and conventional treatments for PTSD patients. We excluded laser acupuncture and acupoint stimulation such as acupressure, moxibustion, tapping, and so forth because of the lack of needling. We excluded trials with controls that acted as “healthy participants.”

#### 2.2.4. Types of Outcome Measures

The most recent guideline for treatment of PTSD [5] includes the following major outcomes: 1st: “reduction in severity of PTSD symptoms”; 2nd: “prevention/reduction of trauma-related comorbid conditions”; 3rd: “patient adherence to treatment plan”; 4th:“response to treatment”; 5th: “social, occupational, adaptive, and interpersonal functioning”; 6th: “quality of life” and 7th: “rate of relapse.” The main outcome measures were any relevant PTSD scales as clinician-administered PTSD scale (CAPS), depression scale, and anxiety scale. Other scales as related to impairment, proportion of patients recovered were extracted following predefined protocol.

### 2.3. Screening, Data Extraction, and Quality Assessment

After screening titles and abstracts retrieved through our search, we excluded all articles that did not match our inclusion/exclusion criteria according to the predefined eligibility criteria mentioned above. Then, expected inclusions were carefully read in full text, and final inclusion was decided by two independent reviewers (Y. D. Kim, I. Heo) by matching method. If studies were written in languages incomprehensible for the reviewers, all articles not written in native language were translated by colleagues. Then we first classified these by the eligibility criteria. If there was a need for full text review, we evaluated these after translation. Data were extracted independently based on predefined characteristics to describe each study (refer to Table 1) by the two reviewers. All disagreements were resolved by discussion and consensus, or by the first author. The Cochrane risk of bias for assessing the quality of included RCTs [22], the CONSORT 2010 checklist for reporting quality of RCTs [23] and the revised standards for reporting interventions in clinical trials of acupuncture (STRICTA) guideline for reporting quality of acupuncture trials [24] were used to evaluate the methodological quality of the included publications. All reviewers were fully trained in the quality assessment and data extraction methodology. 

### 2.4. Data Synthesis and Statistics

Two authors (Y. D. Kim, B. C. Shin) calculated effect estimates (effect size: ES) to summarize the effects of acupuncture on each outcome by recalculation for mean and standard deviation (SD) because all original data were continuous ones. The standardized mean difference (SMD) and 95% confidence interval (CI) on each outcome measurement were calculated using Cochrane Collaboration software (Review Manager (RevMan) Version 5.1.7 for Windows. Copenhagen: The Nordic Cochrane Centre). For meta-analysis, we pooled data across studies using weighted mean difference (WMD) because same measurement was used. Random effect model was used because clinical heterogeneities were expected across the studies. To assess the heterogeneity among the trials, Chi-square test and the Higgins *I* ^2^ test were used.

## 3. Results

### 3.1. Study Description

**Figure 1:**
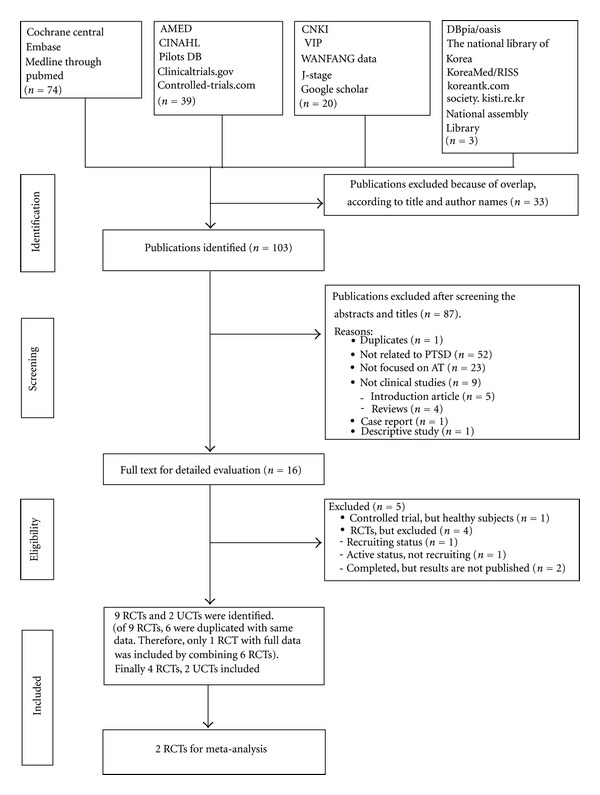
Flow chart of the trial selection process. PTSD: posttraumatic stress disorder; RCT: randomized controlled trial; UCT: uncontrolled clinical trial; AT: acupuncture.

The searches retrieved 136 potentially relevant articles. After screening the titles and abstracts, we excluded 120 studies (Figure 1). 16 articles were read in full and evaluated. Subsequently, 5 studies were excluded because 1 was a controlled trial but the control group members were healthy subjects [30], 1 was active status not recruiting [31], 1 was recruiting status [32], and 2 were completed but with the results not published [33, 34]. Finally 9 RCTs and 2 UCTs were identified. Of 9 RCTs published, Zhang et al. RCT [25] was split or duplicated published with same data [25, 35–39]. So we included only 1 RCT with full data [25] from the 6 RCTs [25, 35–39]. Consequently, 4 RCTs [18, 25–27] and 2 UCTs [28, 29] met our inclusion criteria. Figure 1 sums up the search results based on a four-phase flow diagram in Preferred Reporting Items for Systematic Reviews and Meta-Analyses (PRISMA) statement format [40]. The key data are summarized in Table 1. One RCT originated from the USA [18], while all the others were from China [25–29]. All RCTs adopted a parallel-group design. Two of them were two-parallel-arm group designs [26, 27], one was a three-parallel-arm group design [18], and one was a four-parallel-arm group design [25]. Two RCTs [18, 25] were based on a sample size calculation, whereas the other two RCTs [26, 27] did not report this.

The four RCTs evaluated 543 PTSD patients (mean sample size per arm: 49). The duration of treatment was 1 to 12 weeks. A table showing baseline clinical characteristics for each group was reported in only one RCT [18].

### 3.2. Interventions

One RCT compared needle acupuncture to cognitive-behavioral therapy (CBT) and a waitlist control [18], and another used electroacupuncture only or with moxibustion or with auricular acupuncture versus oral selective serotonin reuptake inhibitors (SSRIs) [25]. One RCT tested electroacupuncture plus moxibustion versus oral SSRI [26], and one RCT compared acupoint stimulation plus CBT to CBT alone [27]. One UCT [29] used just acupuncture, the other UCT [28] used electroacupuncture plus auricular acupuncture and moxibustion. 1 RCT [18] and 1 UCT [29] used manual stimulation without electrical stimulation, and the other 3 RCTs [25–27] and 1 UCT [28] used electrical stimulation. 

### 3.3. Outcomes

#### 3.3.1. Acupuncture versus CBT/Acupuncture versus Waitlist Control/CBT versus Waitlist Control

One high-quality RCT evaluated the effect of acupuncture against CBT and a waitlist control [18]. No statistical difference was found between acupuncture and CBT. But, acupuncture treatment was statistically superior to waitlist control on four outcome measures; posttraumatic symptom scale-self report (PSS-SR) (ES, −0.98; *P* = 0.001), Depression: self-rated Hopkins symptom checklist-25 (HSCL-25) (ES, −0.68; *P* = 0.02), Anxiety: HSCL-25 (ES, −0.91; *P* = 0.003), and Impairment: Sheehan Disability Inventory (SDI) (ES, −0.64; *P* = 0.03, Table 1). The CBT was also statistically superior to waitlist control on four outcome measures; PSS-SR (ES, −0.85; *P* = 0.004), Depression: HSCL-25 (ES, −0.80; *P* = 0.008), Anxiety: HSCL-25 (ES, −0.79; *P* = 0.008), Impairment (ES, −0.64; *P* = 0.03). The therapeutic effects of acupuncture and CBT were similar on the ESs [41] (Table 1).

#### 3.3.2. Acupuncture versus Oral SSRI

One RCT evaluated the effect of electroacupuncture versus oral SSRI [25]. No statistical difference was found between two groups. 

#### 3.3.3. Acupuncture Plus CBT versus CBT Alone

One RCT assessed the effect of acupoint stimulation plus CBT in comparison to CBT alone [27]. Recalculation of the mean difference (MD) revealed a favorable effect of acupoint stimulation plus CBT in terms of IES-R (ES, −1.56; *P* < 0.00001) and the self-compiled questionnaire (ES, −0.59; *P* = 0.01) (Table 1).

#### 3.3.4. Acupuncture Plus Moxibustion versus Oral SSRI

Two RCTs assessed the effects of electroacupuncture plus moxibustion against oral SSRI [25, 26]. One RCT reported no statistical difference between the two groups [25]. However, the other RCT showed that electroacupuncture plus moxibustion was statistically superior to oral SSRI on outcome clinician-administered PTSD scale (CAPS) (ES, −1.77; *P* < 0.00001), depression (ES, −1.96; *P* < 0.00001), and anxiety (ES, −1.53; *P* < 0.00001) [26] (Table 1). 

The meta-analysis of electroacupuncture plus moxibustion versus oral SSRI showed a significant favorable effect of electroacupuncture plus moxibustion on outcome CAPS (2 studies, *n* = 115, ES, −3.19; 95% CI: −3.93 to −2.46, *P* < 0.00001, heterogeneity: *χ* ^2^ = 0.50, *P* = 0.48, *I* ^2^ = 0%), depression (2 studies, *n* = 115, ES, −1.76; 95% CI: −2.21 to −1.31, *P* < 0.00001, heterogeneity: *χ* ^2^ = 1.04, *P* = 0.31, *I* ^2^ = 4%), and anxiety (2 studies, *n* = 115, ES, −1.14; 95% CI: −1.44 to −0.84, *P* < 0.00001, heterogeneity: *χ* ^2^ = 0.62, *P* = 0.43, *I* ^2^ = 0%) (Table 4).

#### 3.3.5. Acupuncture Treatment in 2 UCTs

Two UCTs evaluated acupuncture treatment for total 103 earthquake-caused PTSD patients and showed effectiveness of 94.2% [28] and 91.2% [29], respectively (Table 1).

#### 3.3.6. Adverse Events

Of all 6 studies, 2 RCTs described adverse events related to needle acupuncture [18, 25]. One study noted that some patients (original paper did not report the exact number) mentioned roughness of operational practices, fear of needles, bleeding, hematoma, pain, and fainting [25]. Another study reported just one perceived adverse effect (kidney pain) as a reason for withdrawal from acupuncture treatment [18]. No serious adverse events were reported.

### 3.4. Risk of Bias and Reporting Quality

#### 3.4.1. Risk of Bias in Included RCTs Based on Cochrane Criteria

The risk of bias was low in one RCT [18], whereas one trial [25] had a moderate risk of bias and two trials [26, 27] had a high risk of bias in most categories (Table 2). Two RCTs employed adequate sequence generation methods and allocation concealment [18, 25], whereas the other two [26, 27] failed to report those categories. Assessor blinding was reported in the former two RCTs [18, 25]. The risk of bias for incomplete outcome data was low in only one RCT [18]. In all, the four included RCTs had an unclear risk of bias in terms of selective reporting and other sources of bias. 

#### 3.4.2. Reporting Quality of 4 Included RCTs Based on CONSORT 2010 Checklist

Many leading medical journals and major international editorial groups have endorsed the CONSORT statement, and the statement facilitates critical appraisal and interpretation of RCTs [23]. For this reason, the current review assessed the reporting quality of included RCTs based on the CONSORT 2010 guideline. The 4 included RCTs described 22 items (59.5%) [18] among 37 items, 15 items (40.5%) [25], 9 items (24.3%) [26], and 8 items (21.6%) [27] according to the CONSORT 2010 checklist [23].

#### 3.4.3. Reporting Quality of 4 Included RCTs Based on Revised STRICTA

The STRICTA reporting guideline is an extension of CONSORT was designed to improve the completeness and transparency of reporting of interventions in controlled trials of acupuncture [24], so that such trials may be more accurately interpreted and readily replicated [24]. The reporting quality of acupuncture was high for two of the included RCTs [18, 25], medium in one [26], and low in remaining RCT [27]. The 4 included RCTs reported 16 of 17 items (94.1%) [18], 15 items (88.2%) [25], 13 items (76.5%) [26], and 8 items (47.1%) [27] according to the revised STRICTA guideline. The two high-quality trials [18, 25] presented almost all items transparently except one or two items, whereas the low-quality trial [27] did not describe clearly even the reported 8 items (Table 3).

## 4. Discussion

This is the first systematic review and meta-analysis of prospective clinical trials on the effectiveness of acupuncture for treatment of PTSD. Only 4 RCTs and 2 UCTs met the inclusion criteria for this review. Our main finding of this review is that acupuncture is effective for PTSD based on one high-quality RCT [18] and a meta-analysis. 

The high-quality RCT showed that acupuncture had statistically significant effects compared to a waitlist control, although no statistical difference was found between acupuncture and CBT. Also the therapeutic effect of acupuncture was similar with CBT therapy based on the trial. Additionally, the clinical improvement related to acupuncture or CBT lasted for at least 3 months after the end of treatment in the high-quality RCT.

The meta-analysis showed that acupuncture plus moxibustion was superior to oral SSRI for PTSD. But, we should interpret these results with caution because the meta-analysis was based on one medium-quality RCT [25] and one low-quality RCT [26]. 

One RCT [27] showed that acupoint stimulation plus CBT was more effective than CBT alone in reducing PTSD symptoms. However, acupuncture treatment was not described transparently. Therefore, this result had doubtful reliability. 

We found a similar pattern of reporting quality when comparing the Cochrane risk of bias [22] with the CONSORT 2010 checklist [23]. Two of the included studies [18, 25] had a high reporting quality in terms of acupuncture based on the revised STRICTA guideline [24]. All the studies failed to describe in detail adverse effects related to acupuncture.

We would like to emphasize the clinical importance of acupuncture for PTSD. Acupuncture might be useful in emergency medicine [44]. A recent case series study suggested possible effectiveness of acupuncture in emergency conditions involving PTSD and emotional trauma [45]. In addition, acupuncture is a conveniently portable medical device for taking emergency measures, and it is very cheap, safe, and easy to handle for trained practitioners. 

According to a study [46], during long-term SSRI therapy, the most troubling adverse effects were sexual dysfunction, weight gain, and sleep disturbance. The incidence rate of sexual dysfunction was reported as 2% to 7% [47]. Mean weight gain of 10.8 kg (24 lbs) was found after 6 to 12 months of paroxetine therapy [48]. On the other hand, for acupuncture, “mild” adverse events of such as bleeding, bruising, pain on needling occurred in rate of 6.8% (2,178 out of 31,822 sessions) [49]. And no serious adverse events were reported in total 66,229 treatment sessions according to two studies [49, 50]. Therefore acupuncture may be a relatively safe alternative for PTSD in contrast to SSRI, if long-term therapy is needed for treatment.

This systematic review has several limitations. First, although we made strong efforts to retrieve all RCTs on the subject, the evidence reviewed is potentially incomplete because only one rigorous study was included. Second, because there was no RCT on PTSD with a sham acupuncture control, we could not evaluate the effects of acupuncture compared to an inert placebo control [51]. Third, study design was quite different across the four included RCTs. Two RCTs [18, 25] compared acupuncture with different controls (CBT and oral SSRI), and the other two RCTs [26, 27] employed acupuncture as a cointervention of moxibustion and CBT. These very different designs across studies prevented us from abstracting a firm conclusion. Furthermore, the paucity of included trials and the suboptimal methodological quality of the primary data overall, except for one high-quality trial, are also important vulnerabilities of this review. 

In total, from these drawbacks we could suggest several important recommendations for future research in this area. One is a need for appropriate controls such as sham/placebo control or other relevant active controls for testing the efficacy or effectiveness of acupuncture for PTSD in the design of parallel RCT or comparative effectiveness research. The second is outcomes should be used by validated one as primary one is PTSD scale and the secondary one is depression or anxiety with safety reporting. The third is high methodological quality is strongly required, as adequate randomization with allocation concealment, blinding of participants and assessors, or sample size estimation for power of trial, with following guideline of CONSORT and STRICTA.

## 5. Conclusions

The results of this systematic review and meta-analysis suggest that evidence of the effectiveness of acupuncture for PTSD is encouraging but not cogent, because only two RCTs were included in meta-analysis, and it is too small to verify the efficacy of acupuncture. For the future researches, sham-controlled RCTs [52] or comparative effectiveness researches [53] are required to test efficacy and effectiveness of acupuncture for PTSD. To prevent performance bias and detection bias, blinding of participants and outcome assessment should be kept in future trials, too.

## Figures and Tables

**Table 1:** Summary of randomized controlled trials and prospective clinical trials of acupuncture for posttraumatic stress disorder.

First author [ref] (year) country	Population	Study design	Sample size/*N*, analyzed	Intervention/control group (regime)	Treatment session	Main outcomes	Intergroup difference	Comments
RCT (*n* = 4)								

Hollifield [18] (2007) U.S.A.	28 out of 84 identified childhood abuse/ Others, unknown trauma	3 arm parallel, open	84/73	(A) AT + AAT (*n* = 29)/ (B) CBT (*n* = 28) (C) WLC (*n* = 27)	24 sessions	(1) PTSD scale (PSS-SR) (2) Depression (HSCL-25) (3) Anxiety (HSCL-25) (4) Impairment (SDI)	(1) A versus B: *P* = 0.36, MD, −0.26 [−0.83, 0.30] A versus C: *P* = 0.001, MD, −0.98 [−1.58, −0.38] B versus C: *P* = 0.004, MD, −0.85 [−1.44, −0.27] (2) A versus B: *P* = 0.92 MD, 0.03 [−0.53, 0.59] A versus C: *P* = 0.02, MD, −0.68 [−1.27, −0.10] B versus C: *P* = 0.008, MD, −0.80 [−1.38, −0.21] (3) A versus B: *P* = 0.39, MD, −0.25 [−0.81, 0.31] A versus C: *P* = 0.003, MD, −0.91 [−1.51, −0.32] B versus C: *P* = 0.008, MD, −0.79 [−1.37, −0.21] (4) A versus B: *P* = 0.98, MD, −0.01 [−0.57, 0.55] A versus C: *P* = 0.03, MD, −0.64 [−1.22, −0.06] B versus C: *P* = 0.03, MD, −0.64 [−1.22, −0.07]	The AT group had significantly better improvements in PTSD symptoms than the WLC group. But, there was no statistically significant difference between the AT group and the CBT group.
Zhang [25] (2010)China	Earthquake	4 arm parallel, open	276/256	(A) EA (*n* = 69) (B) EA + moxa (*n* = 69) (C) EA + AAT (*n* = 69)/ (D) Oral SSRI (*n* = 69)	36 sessions	(1) PTSD scale (CAPS) (2) Depression (HAMD) (3) Anxiety (HAMA)	(1) A versus D: *P* = 0.43, MD, −0.13 [−0.47, 0.20] B versus D: *P* = 0.88, MD, −0.03 [−0.36, 0.31] C versus D: *P* = 0.55, MD, −0.10 [−0.44, 0.23] (2) A versus D: *P* = 0.14, MD, −0.25 [−0.59, 0.08] B versus D: *P* = 0.34, MD, −0.16 [−0.50, 0.17] C versus D: *P* = 0.23, MD, −0.21 [−0.54, 0.13] (3) A versus D: *P* = 0.34, MD, −0.16 [−0.50, 0.17] B versus D: *P* = 0.64, MD, −0.08 [−0.41, 0.25] C versus D: *P* = 0.54, MD, 0.11 [−0.23, 0.44]	The therapeutic effect of EA was not better than that of oral SSRI.
Zhang [26](2010) China	Earthquake	2 arm parallel open	92/81	(A) EA + moxa (*n* = 46)/ (B) Oral SSRI (*n* = 46)	36 sessions	(1) PTSD scale (CAPS) (2) Depression (HAMD) (3) Anxiety (HAMA)	(1) A versus B: *P* < 0.00001, MD, −1.77 [−2.26, −1.29] (2) A versus B: *P* < 0.00001, MD, −1.96 [−2.46, −1.46] (3) A versus B: *P* < 0.00001, MD, −1.53 [−2.00, −1.07]	EA plus moxa was more effective than oral SSRI therapy.
Zhang [27] (2011) China	Earthquake	2 arm parallel open	91/90	(A) Acupoint Stimulation + CBT (*n* = 67)/ (B) CBT (*n* = 24)	3~4 sessions*	(1) PTSD scale (IES-R) (2) PTSD scale (self compiled questionnaire)	(1) A versus B: *P* < 0.00001, MD, −1.56 [−2.08, −1.04] (2) A versus B: *P* = 0.01, MD, −0.59 [−1.07, −0.12]	The acupoint stimulation plus CBT showed better efficacy than CBT therapy alone.

UCT (*n* = 2)								

Wang [28] (2009) China	Earthquake	UCT	69	EA + AAT + moxa	36 sessions	(1) The number of cured/improved/non-improved	Not applicable	Treatment was effective in 65 out of 69 (94.2%).
Yuan [29] (2009) China	Earthquake	UCT	34	AT	20 sessions	(1) The number of cured/improved/non-improved	Not applicable	AT was effective in 31 out of 34 (91.2%).

Abbreviations: RCT: randomized controlled trial; UCT: uncontrolled clinical trial; AT: classical acupuncture; EA: electro-acupuncture; moxa, moxibustion; AAT: auricular acupuncture; CBT: cognitive behavioral therapy; WLC: waitlist control; SSRI: selective serotonin reuptake inhibitors; PSS-SR: posttraumatic symptom scale-self report; HSCL-25: self-rated Hopkins symptom checklist-25; SDI: Sheehan Disability Inventory; MD: mean difference; CAPS: clinician-administered PTSD scale; HAMD: Hamilton depression rating scale; HAMA: Hamilton anxiety rating scale; IES-R: Chinese version of the incident effect scale revised; *treated a time every other day for 1 week.

**Table 2:** Cochrane risk of bias of included randomized controlled trials.

First author [ref] (Year)	Hollifield [18] (2007)	Zhang [25] (2010)	Zhang [26](2010)	Zhang [27] (2011)
(1) Random sequence generation (selection bias)	L (computerized randomization)	L (computerized randomization)	U	U
(2) Allocation concealment (selection bias)	L (central allocation)	L (sequentially numbered, opaque sealed envelopes)	U	U
(3) Blinding of participants (performance bias)	H	H	H	H
(4) Blinding of outcome assessment (detection bias)	L (mentioned)	L (mentioned)	U	U
(5) Incomplete outcome data (attrition bias)	L (mentioned)	U	U	U
(6) Selective reporting (reporting bias)	U	U	U	U
(7) Other sources of bias (other bias)	U	U	U	U

L: low risk of bias; H: high risk of bias; U: unclear.

**Table 3:** Reporting quality of 4 included RCTs based on revised STRICTA.

Checklist item	Hollifield et al. [18] (2007)	Zhang et al. [25](2010)	Zhang et al. [26](2010)	Zhang et al. [27](2011)
(1) Acupuncture rationale				
(1a) Style of acupuncture	TCM	TCM	TCM	n.r.
(1b) Reasoning for treatment provided	A paper by Napadow et al., 2005 [42]	A paper by Hollifield et al., 2007 [18]	A paper by Hollifield et al., 2007 [18]	n.r.
(1c) Extent to which treatment was varied	2 types of AT (1) AT: 25 fixed needles plus up to 3 flexible needles within 15 points (2) AAT: ≥6 vaccaria seeds	Fixed interventions (A) EA only (B) EA + moxa (C) EA + AAT	Fixed interventions EA + moxa	Fixed intervention

(2) Details of needling				
(2a) Number of needle insertions per subject per session	(1) AT: 25 plus up to 3 needles (2) AAT: ≥6 vaccaria seeds	(1) EA: 8 needles (2) AAT: 6 vaccaria seeds	EA: 8 needles	unclear.
(2b) Names of points used	(1) AT: bilateral at LR3, PC6, HT7, ST36, SP6, GB20, BL14, 15, 18, 20, 21 and 23/unilateral at Yintang (2) AAT: unilateral at Shenmen, Sympathetic, Liver, Kidney, Lung points	(1) EA: bilateral at GB20/unilateral at GV24, EX-HN1, GV20 (2) AAT: unilateral at Subcortex, Shenmen, Sympathetic, Heart, Liver, Kidney (3) moxa: bilateral at BL23, BL52/unilateral at GV4	(1) EA: bilateral at GB20/unilateral at GV24, EX-HN1, GV20 (2) moxa: bilateral at BL23, BL52/unilateral at GV4	Unilateral at left PC8
(2c) Depth of insertion	(1) AT: 1/4 to 1/2 inch (2) AAT: not inserted	(1) EA: 0.5 to 1.2 cun (2) AAT: not inserted	EA: 0.5 to 1.2 cun	n.r.
(2d) Responses sought	(1) AT: n.r. (2) AAT: not applicable	(1) EA: de-qi (2) AAT: not applicable.	EA: de-qi	n.r.
(2e) Needle stimulation	(1) AT: manipulation (2) AAT: self-massage on the seeds for 15 min/d	(1) EA: electrical stimulation, 100 Hz (2) AAT: 1-2 min pressure	EA: electrical stimulation, 5~8.3 Hz	A Japanese stimulator with 50 Hz was used
(2f) Needle retention time	(1) AT: 25–40 min (2) AAT: unclear	(A) 30 min (B) 30 min (C) 30 min	30 min	Unclear, but the left PC8 was stimulated for 30 min
(2g) Needle type	(1) AT: Viva needles, 34 g (2) AAT: vaccaria seeds	(1) EA: 0.30 mm × 40 mm (2) AAT: vaccaria seeds	n.r.	n.r.

(3) Treatment regimen				
(3a) Number of treatment sessions	24 sessions	36 sessions	(1) EA: 18 sessions (2) moxa: 36 sessions	3~4 sessions*
(3b) Frequency and duration of treatment sessions	Twice a week, 1 hour per session, 12 weeks	Three times a week, 12 weeks	(1) EA: three times a week, 6 weeks (2) moxa: three times a week, 12 weeks	A time every other day for 1 week

(4) Other components of treatment				
(4a) Details of other interventions administered to the acupuncture group	Patients were taught how to use vaccaria seeds for symptom management	(3) moxa: 30 g and 20 min/session, wooden moxibustion box 20 mm × 15 mm × 12 mm	(2) moxa: 20 min/session	CBT
(4b) Setting and context of treatment	n.r.	n.r.	n.r.	n.r.

(5) Practitioner background				
(5) Description of participating Acupuncturists	Doctor of Oriental Medicine in New Mexico with 4 years postgraduate TCM clinical experience	n.r.	n.r.	n.r.

(6) Control or comparator interventions				
(6a) Rationale for the control or comparator	(B) A review by Bisson and Andrew, 2005 [43] (C) not applicable	Approval of FDA	n.r.	n.r.
(6b) Precise description of the control or comparator	(B) CBT (C) WLC	(D) Oral SSRI (Paroxetine 20 mg, once/day, 12 weeks)	Oral SSRI (Paroxetine 20 mg, once/day, 12 weeks)	(B) CBT

Abbreviations: RCT: randomized controlled trial; TCM: traditional Chinese medicine; n.r: not reported; AT: classical acupuncture; EA: electro-acupuncture; moxa, moxibustion; AAT: auricular acupuncture; CBT: cognitive behavioral therapy; WLC: waitlist control; SSRI: selective serotonin reuptake inhibitors.

*treated a time every other day for 1 week.

**Table:** (a) PTSD scale (CAPS).

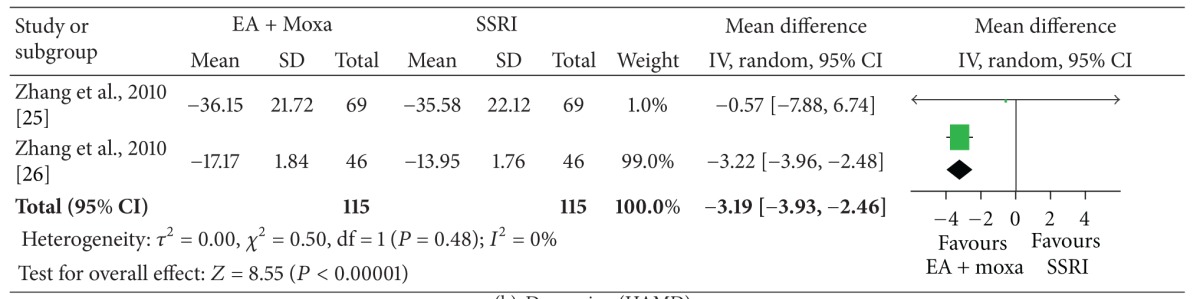

**Table:** (b) Depression (HAMD).

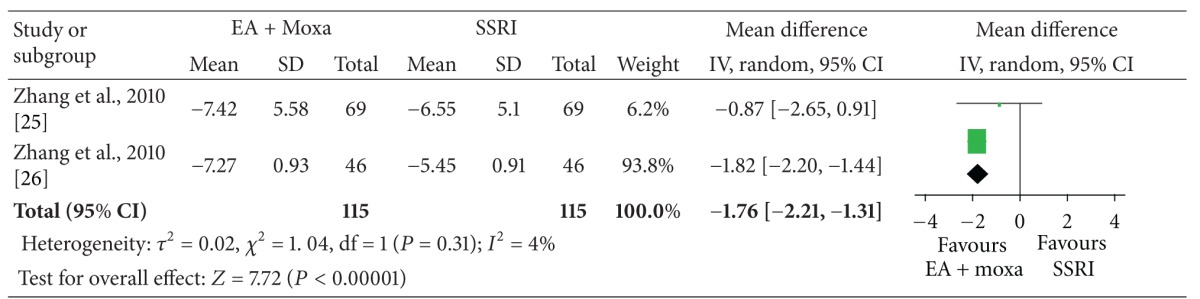

**Table:** (c) Anxiety (HAMA).